# Conditional Deletion of *Hsd11b2* in the Brain Causes Salt Appetite and Hypertension

**DOI:** 10.1161/CIRCULATIONAHA.115.019341

**Published:** 2016-04-04

**Authors:** Louise C. Evans, Jessica R. Ivy, Caitlin Wyrwoll, Julie A. McNairn, Robert I. Menzies, Thorbjørn H. Christensen, Emad A.S. Al-Dujaili, Christopher J. Kenyon, John J. Mullins, Jonathan R. Seckl, Megan C. Holmes, Matthew A. Bailey

**Affiliations:** From British Heart Foundation Centre for Cardiovascular Science, The University of Edinburgh, United Kingdom (L.C.E., J.R.I., C.W., J.A.M., R.I.M., T.H.C., C.J.K., J.J.M., J.R.S., M.C.H., M.A.B.); and Dietetics, Nutrition and Biological Sciences Department, Queen Margaret University, Edinburgh, United Kingdom (E.A.S.Al-D.). The current address for Dr Evans is Department of Physiology, Cardiovascular Center, Medical College of Wisconsin, Milwaukee; the current address for Dr Wyrwoll is School of Anatomy, Physiology & Human Biology, The University of Western Australia, Crawley, Australia; and the current address for Dr Christensen is Department of Cardiovascular and Renal Research, University of Southern Denmark, Odense.

**Keywords:** aldosterone, mineralocorticoids, pressoreceptors, salt, solitary nucleus

## Abstract

**Background—:**

The hypertensive syndrome of Apparent Mineralocorticoid Excess is caused by loss-of-function mutations in the gene encoding 11β-hydroxysteroid dehydrogenase type 2 (11βHSD2), allowing inappropriate activation of the mineralocorticoid receptor by endogenous glucocorticoid. Hypertension is attributed to sodium retention in the distal nephron, but 11βHSD2 is also expressed in the brain. However, the central contribution to Apparent Mineralocorticoid Excess and other hypertensive states is often overlooked and is unresolved. We therefore used a Cre-Lox strategy to generate 11βHSD2 brain-specific knockout (*Hsd11b2*.BKO) mice, measuring blood pressure and salt appetite in adults.

**Methods and Results—:**

Basal blood pressure, electrolytes, and circulating corticosteroids were unaffected in *Hsd11b2*.BKO mice. When offered saline to drink, *Hsd11b2*.BKO mice consumed 3 times more sodium than controls and became hypertensive. Salt appetite was inhibited by spironolactone. Control mice fed the same daily sodium intake remained normotensive, showing the intrinsic salt resistance of the background strain. Dexamethasone suppressed endogenous glucocorticoid and abolished the salt-induced blood pressure differential between genotypes. Salt sensitivity in *Hsd11b2*.BKO mice was not caused by impaired renal sodium excretion or volume expansion; pressor responses to phenylephrine were enhanced and baroreflexes impaired in these animals.

**Conclusions—:**

Reduced 11βHSD2 activity in the brain does not intrinsically cause hypertension, but it promotes a hunger for salt and a transition from salt resistance to salt sensitivity. Our data suggest that 11βHSD2-positive neurons integrate salt appetite and the blood pressure response to dietary sodium through a mineralocorticoid receptor–dependent pathway. Therefore, central mineralocorticoid receptor antagonism could increase compliance to low-sodium regimens and help blood pressure management in cardiovascular disease.

## Abstract 2

Supplemental Digital Content is available in the text.

Congenital, acquired (licorice ingestion), or age-related deficiency in the glucocorticoid-metabolizing enzyme 11β-hydroxysteroid dehydrogenase type 2 (11βHSD2) promotes low-renin hypertension, hypokalemia, and sodium retention attributable to unregulated activation of the mineralocorticoid receptor (MR) by endogenous cortisol (corticosterone in rodents).^1^ Reduced 11βHSD2 activity causes a spectrum of disease: genetic ablation of the enzyme causes the life-threatening syndrome of Apparent Mineralocorticoid Excess (AME; OMIM +218030), diagnosed in early childhood^2^; reduced activity causes hypertension in adults,^3^ and loss-of-function variants in *HSD11B2* are associated with increased blood pressure per se or with salt sensitivity of blood pressure.^4,5^

Editorial, see p 1335

Clinical Perspective on p 1370

AME presents with sodium retention^6^ and, in common with monogenic Liddle syndrome,^7^ can be resolved by renal transplantation.^8^ This suggests that high blood pressure follows the kidney,^9^ at least in these spectral disorders. This renal-centric view of hypertension is supported by our studies in *Hsd11b2* null mice, which are hypertensive on a basal salt intake;^10^ renal sodium excretion is reduced, and sodium transport pathways in the aldosterone-sensitive distal nephron are inappropriately activated.^11,12^ Similarly, *Hsd11b2* heterozygote null mice, which have normal basal blood pressure, cannot efficiently excrete a sodium load and are salt sensitive.^13,14^

11βHSD2 is also normally expressed in the brain, but the contribution of central pathways to hypertension in AME and other hypertensive states is poorly understood and often overlooked. Studies in humans suggest that 11βHSD2 in the brain may contribute to abnormal sodium homeostasis: increased salt appetite has been reported in AME^15^ and loss-of-function variants positively associate with sodium intake in the general population.^16^ Moreover, the sympathetic nervous system is activated in *Hsd11b2* null mice, contributing importantly to the maintenance of hypertension in these animals.^11^

11βHSD2 has a widespread central expression during fetal development and modulates glucocorticoid programming of adult behavior and cognitive function.^17^ Fetal 11βHSD2 expression is progressively silenced from midgestation, and, in adulthood, 11βHSD2 is restricted to subpopulations of neurons in brain areas influencing blood pressure and, less certainly, salt appetite.^17–19^ In the adult mouse, *Hsd11b2* is only expressed in the nucleus of the solitary tract (NTS).^20^ However, defining the role of 11βHSD2 in these NTS neurons of the adult brain has been challenging. Overstimulation of these neurons by intracerebrovascular infusion of aldosterone^21^ or 11βHSD2 inhibitors^22^ increases blood pressure. Such studies are informative but lack precision; conventional gene targeting induces a complex and unstable phenotype with deranged systemic electrolyte and hormonal status.^11^ We therefore recently used a Cre-Lox strategy to conditionally delete *Hsd11b2* in the mouse central nervous system. At embryonic day 12.5, the peak of gestational 11βHSD2 expression in the brain, mRNA abundance was reduced by 96% in the knockout mice.^23^ This programmed depressive behavior and cognitive impairment in adulthood.^23^ Renal 11βHSD2 expression was not affected by conditional brain targeting, and, in adults, basal blood pressure and sodium excretion were normal.^23^ In the current study, we show that central deletion of *Hsd11b2* causes an innate salt appetite, leading to a sustained increase in blood pressure without systemic sodium retention. Hypertension was associated with an exaggerated pressor response to α-adrenoreceptor activation and an attenuated baroreflex.

## Methods

### Generation of Experimental Mice

*Hsd11b2*^f/f^ mice were generated on a C57BL6 background (Artemis Pharmaceuticals, Cologne, Germany) by inserting LoxP sites into introns 1 and 5. These mice were bred with transgenic mice expressing Cre recombinase under the control of a rat nestin promoter/enhancer (B6.Cg-Tg(Nes-cre)1Kln/J; Jackson Laboratory, Bar Harbor, ME), as we described.^23^ This generated Nestin-Cre.*Hsd11b2*^fl/fl^ offspring (*Hsd11b2* Brain Knockout; *Hsd11b2*.BKO) and *Hsd11b2*^fl/fl^ littermate controls. All experiments were performed blinded to genotype and in accordance with the United Kingdom Home Office Animals (Scientific Procedures) Act, following ethical review by the University.

### Measurement of 11βHSD2 Expression and Activity

mRNA abundance for *Hsd11b2* in whole kidney and in isolated NTS was assessed by quantitative polymerase chain reaction and quantified by using the second derivative maximum method.^24^ 11βHSD2 expression in the aldosterone-sensitive distal nephron was confirmed by immunohistochemistry, and 11βHSD2 enzyme activity was measured as the conversion of [^3^H]corticosterone to [^3^H]dehydrocorticosterone, quantified by thin-layer chromatography.

### Blood Pressure Measurement

Radiotelemetry devices (model TA-11PAC-10, Data Systems International, St Paul, MN) were inserted into *Hsd11b2*.BKO (n=6) and control mice (n=6) under ketamine-medetomidine anesthesia. After a week of postoperative recovery, data were collected over a 5-minute period every 20 minutes at an acquisition rate of 2 kHz. Mice were housed under controlled temperature (21±1°C) and humidity (50±10%) with a fixed 12-hour light:dark cycle (lights on 7 am local time). Each animal underwent the following protocols.

#### Ad Libitum Salt Intake

Blood pressure was recorded over a 7-day baseline period during which mice were able to drink from 2 bottles containing deionized water. This experiment was repeated in an independent cohort of nontelemetered mice, and the data sets were merged to give Hsd11b2.BKO (n=12) and control (n=9). Water intake was ≈4 mL/24 h and was not different between groups. After 7 days, 1 water bottle was replaced with a 1.5% NaCl bottle for a 21-day period. Bottle position was alternated every 24 hours to negate side preference. Throughout this experiment, both groups of mice had a similar food intake.

#### Fixed Salt Intake

Mice were fed a diet in which sodium was incorporated as a powdered chow mixed with gelatin. During baseline, the diet contained ≈0.1% sodium by weight, which was then increased to ≈1% sodium for a 7-day period. The amount of the gel consumed per day was predetermined to ensure that mice ate the entire block, clamping sodium intake across genotypes during the experimental phase. Mice had access to deionized drinking water throughout this experiment, and blood pressure was recorded by radiotelemetry.

#### Dexamethasone

Once blood pressure had reached steady state under matched sodium feeding, dexamethasone (DEX) was administered via the drinking water (1 μg/mL in 0.1% ethanol) and plasma corticosterone measured at 7 pm was reduced in both genotypes (*Hsd11b2*.BKO=186±38 versus 31±5 nmol/L after DEX; Control=205±18 basal versus 43±8 nmol/L after DEX).

### Salt-Taste Threshold

In a cohort of control (n=4) and *Hsd11b2*.BKO (n=4) mice, taste threshold was assessed by offering a first drinking bottle containing deionized water and a second containing either a saline solution (0.25%–3%) or quinine (1%). Each measurement was made over 48 hours.

### Mineralocorticoid Receptor Antagonism

Intake of 1.5% saline was determined in a separate group of Hsd11b2.BKO mice (n=8), before (baseline) and after MR antagonism with spironolactone; measurements were also made in a group (n=3) of control mice. Spironolactone was distributed 1:4 w:w in an elastomer matrix (Silastic MDX4-4210, Dow Corning) and pellets cured overnight at 37°C. After ad libitum salt preference had been measured, pellets were implanted subcutaneously under isoflurane anesthesia. Each pellet contained ≈30 mg of the drug, designed to achieve a plasma concentration of canrenone (the active metabolite of spironolactone) of ≈75 nmol/L.^25^

### Sodium Balance in Conscious Mice

Mice (n=6 of each genotype) were housed in individual metabolism cages for measurement of sodium and potassium excretion, first on basal sodium diet (0.1% sodium), then 1% sodium diet. Urinary sodium and potassium concentration was measured by flame photometry; plasma sodium and potassium were measured by ion-selective electrode (AVI 9180 Electrolyte analyzer, Roche UK). Aldosterone^26^ and corticosterone^27^ concentration in urine was measured by enzyme-linked immunosorbent assay.

### Baroreceptor Reflex

The integrated baroreceptor reflex was assessed pharmacologically in anesthetized mice (thiobutabarbital; 120 mg/kg IP) maintained on either 0.1% sodium diet or 1% sodium diet for 7 days before the experiment. A cannula was inserted into the jugular vein and a tracheostomy was performed. A cannula filled with heparin-saline was placed in the carotid artery. The cannula was made from a ≈5-mm length of p10 Portex tubing inserted into a ≈50-mm length of p50 tubing. The undampened pulse wave was recorded continuously at 1 kHz using a Capto pressure transducer connected to a Powerlab (AD Instruments, Oxford, UK). After postsurgical equilibration, sodium nitroprusside (30, 60, and 120 μg/kg) and phenylephrine (10, 20, and 40 μg/kg) were injected intravenously in random order, to induce acute decreases and acute increases in blood pressure, respectively. For each injection, the change in heart rate at the peak change in systolic blood pressure (SBP) was recorded and Δheart rate/ΔSBP was used as an index of baroreceptor gain.

### Statistics

Data are presented as mean±standard error, as medians with interquartile range, or as linear regression with 95% confidence interval, as appropriate. Statistical comparisons (Graphpad Prism 6, La Jolla, CA) were made by using 2-way analysis of variance (ANOVA) with repeated measures, Mann-Whitney *U* or *t* tests, as stated in the figure legends. For 2-way ANOVA, we assessed the main effects of the genotype and treatment and the interaction between the 2. When used, planned or post hoc comparisons were made by using Holm-Sidak test to correct for multiple comparisons. The family *P* value was fixed at 0.05, and the number of comparisons is indicated in the figure legends. The diurnal variation in SBP and heart rate was characterized by cosinor analysis,^28^ calculating by sine function least-squares regression, mesor, amplitude, and acrophase for each mouse; these values were then used to calculate the group mean comparison between genotypes by the Welch *t* test. The goodness-of-fit model was confirmed in all cases by the significance of the *F* statistic using the zero-amplitude test (*P*<0.01 or less).

## Results

### Baseline Parameters

The expression of *Hsd11b2* mRNA in the NTS of adult *Hsd11b2*.BKO mice was reduced by >90% in comparison with controls (Figure I in the online-only Data Supplement). Expression and localization of renal 11βHSD2 in adult *Hsd11b2*.BKO mice was not different from control animals (Figure II in the online-only Data Supplement).

Under baseline conditions SBP, diastolic blood pressure (DBP), and heart rate were similar in *Hsd11b2*.BKO mice and controls (Figure III in the online-only Data Supplement; Table I in the online-only Data Supplement); the acrophase of the diurnal variation for SBP and heart rate corresponded to 3 am local time in both groups of animals. Food/water intake, plasma electrolytes, hematocrit, and corticosteroids were not different between genotypes (Table II in the online-only Data Supplement). These data contrast with observations in animals with global *Hsd11b2* deletion,^10,11^ which are hypertensive and hyperkalemic and have a suppressed renin-angiotensin-aldosterone system under conditions of basal sodium intake.

### Salt-Sensitive Hypertension in *Hsd11b2*.BKO Mice

**Figure 1.**
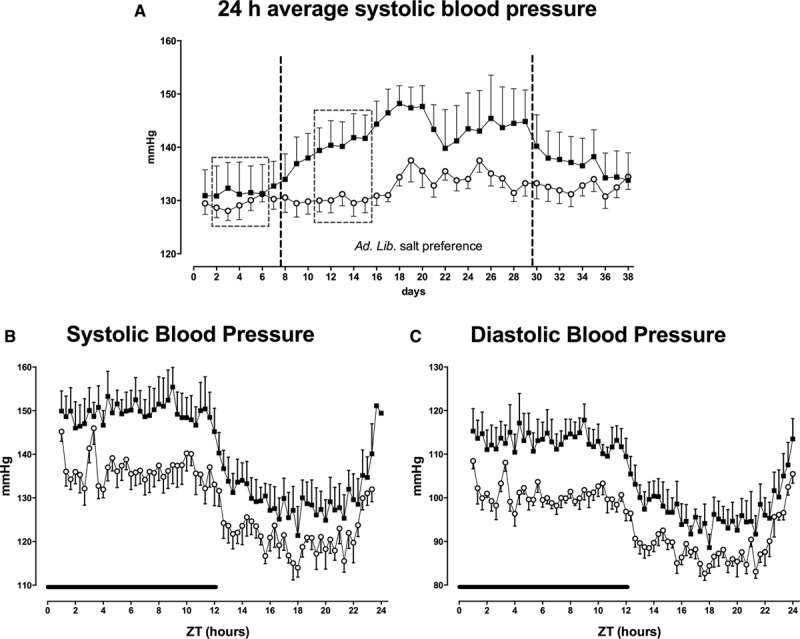
Salt sensitivity in *Hsd11b2*.BKO mice. Blood pressure was measured in conscious, unrestrained *Hsd11b2*.BKO (n=6; filled squares) and control mice (n=6; open circles) using radiotelemetry. All mice had access to 2 drinking bottles for the entire experiment; from day 8 to 29, 1 bottle contained 1.5% NaCl; at other times, both bottles contained water. **A**, 24-hour average systolic blood pressure. Data are mean±SEM. Two-way ANOVA reported a significant effect of genotype (*P*<0.0001), of treatment (*P*=0.013), and of the interaction between the main effects (*P*=0.0021). Mesor, amplitude, and acrophase were calculated by cosinor analysis (Figure I and Table II in the online-only Data Supplement) of nonaveraged data obtained over consecutive days indicated by the boxes. Systolic blood pressure (**B**) and diastolic blood pressure (**C**) measured every 20 minutes over a 24-hour period. The black line indicates subjective night (7 pm to 7 am local time). Data are group mean±SEM, generated by averaging each mouse over 5 consecutive days of recording. Mesor, amplitude, and acrophase were calculated by cosinor analysis (Table II in the online-only Data Supplement). ANOVA indicates analysis of variance; and SEM, standard error of the mean.

When offered 1.5% NaCl solution to drink, *Hsd11b2*.BKO mice became hypertensive, average 24-hour SBP increasing by 20 to 30 mm Hg over a 2-week period (Figure 1A); blood pressure was not changed in control mice during ad libitum access to saline. In *Hsd11b2*.BKO mice, blood pressure returned to baseline when the saline-drinking option was withdrawn (Figure 1A).

Cosinor analysis was performed on data acquired over 4 consecutive days (periods indicated in Figure 1A) during both basal and saline periods. High salt intake caused a significant increase in mesor SBP in *Hsd11b2*.BKO mice but not in controls (Figure IVA in the online-only Data Supplement; Table I in the online-only Data Supplement). The amplitude of the diurnal SBP variation was also significantly higher in *Hsd11b2*.BKO mice than in controls (Figure IVB in the online-only Data Supplement; Table I in the online-only Data Supplement), whereas acrophase was not affected by sodium intake. Both SBP (Figure 1B) and DBP (Figure 1C) were significantly elevated during the dark phase of the day/night cycle in *Hsd11b2*.BKO mice, but this salt sensitivity was not associated with a genotypic difference in the heart rate over the 24-hour cycle (Figure V in the online-only Data Supplement; Table I in the online-only Data Supplement).

### Salt Appetite and Hypertension

**Figure 2.**
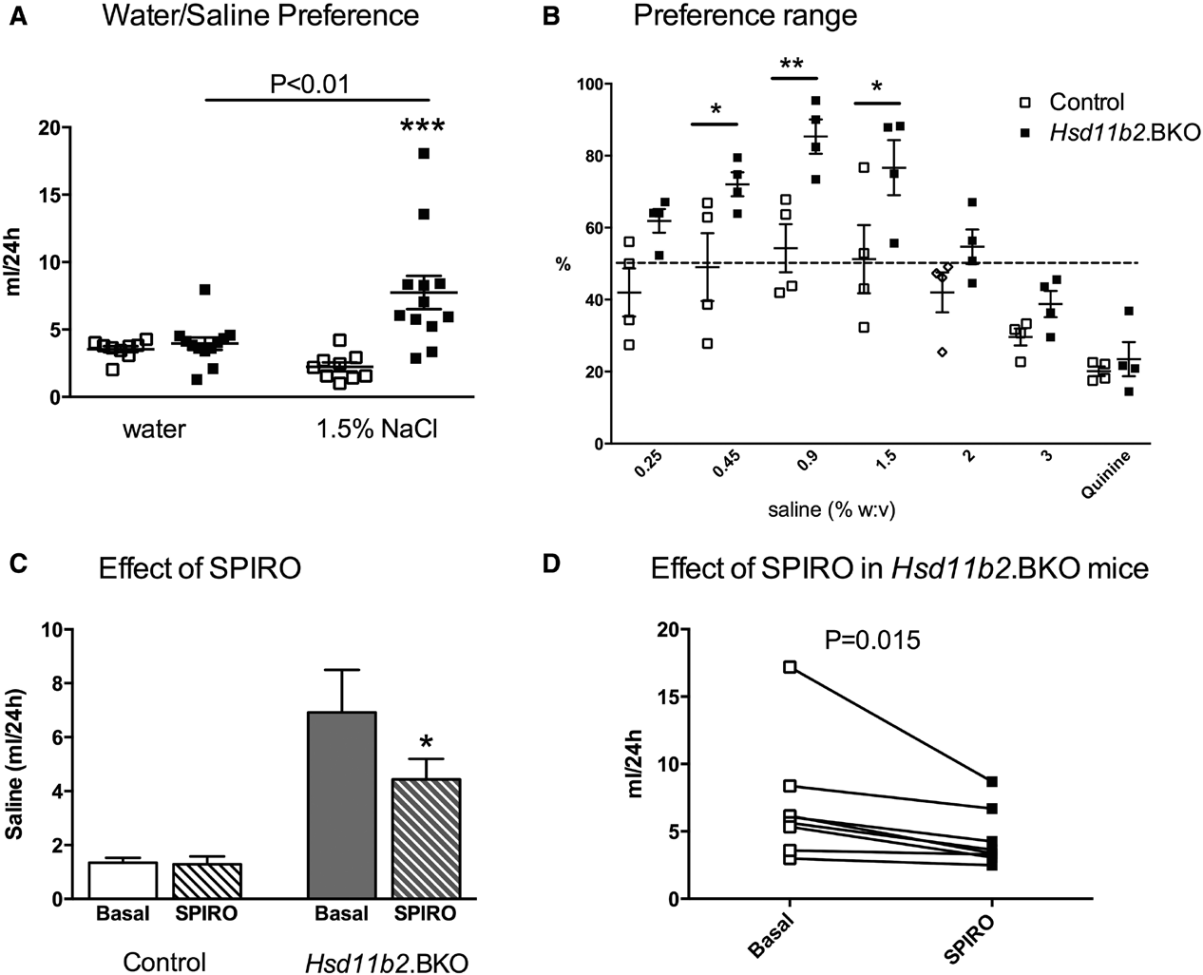
Salt-appetite in *Hsd11b2*.BKO mice. **A**, Water and 1.5% saline intake per 24 hours in *Hsd11b2*.BKO (gray bars; n=12) and controls (open bars; n=9) mice. Individual data and group mean±SEM are shown. Two-way ANOVA indicates a significant effect of genotype (*P*=0.002) and interaction between genotype and drinking behavior (*P*=0.002). Two post hoc comparisons were made, *P* values as indicated. ***P<0.001. **B**, Preference testing for 0.25% to 3% saline and quinine versus water in *Hsd11b2*.BKO (gray bars; n=4) and controls (open bars; n=4) mice. The dashed line indicates no preference, and values below this line indicate aversion. Individual data and group mean±SEM are shown. Two-way ANOVA reported a significant effect of genotype (*P*<0.0001). Six multiple comparisons were made and *P* values are as indicated. **P<0.01, *P<0.05. **C**, 1.5% saline intake in *Hsd11b2*.BKO (n=8) and control mice before (open bars) and after systemic spironolactone treatment (hashed bars). Group mean±SEM are shown. **D**, Effect of spironolactone (filled squares) on basal salt intake (open squares) in *Hsd11b2*.BKO mice in comparison with 1-tailed paired *t* test. ANOVA indicates analysis of variance; SEM, standard error of the mean; and SPIRO, spironolactone.

Both *Hsd11b2*.BKO and control mice had a daily deionized water intake of ≈4 mL. When presented with the option, *Hsd11b2*.BKO mice spontaneously drank ≈8 mL/24 h of 1.5% NaCl while maintaining their deionized water intake (Figure 2A). *Hsd11b2*.BKO mice had salt preference, saline accounting for >60% of total fluid intake. Control mice also drank from the saline bottle but displayed a modest salt aversion, with saline accounting for <40% of total intake. Thus, daily sodium intake increased significantly in both genotypes, but the average intake over the experiment was ≈3 times higher in the *Hsd11b2*.BKO mice than in controls (*Hsd11b2*.BKO=3154±352 μmol/24 h; Control=982±129 μmol/24 h; *P*<0.001).

We were not able to detect a lower threshold for salt preference, *Hsd11b2*.BKO mice maintained a higher saline-to-water intake at all but the highest concentration (3% NaCl) tested (Figure 2B). This abnormality was not a generalized taste phenomenon, because *Hsd11b2*.BKO mice retained an aversion for quinine (Figure 2B). Systemic administration of the MR antagonist, spironolactone, did not affect saline intake in the 3 control mice (Figure 2C) but reduced saline drinking in all 8 *Hsd11b2*.BKO mice tested (Figure 2D). On average, spironolactone reduced saline intake to 69±5% of predrug values (*P*=0.0006, 1-sample *t* test). Nevertheless, saline intake remained higher in *Hsd11b2*.BKO mice than in controls during spironolactone treatment. Spironolactone did not affect water consumption in either group of mice.

**Figure 3.**
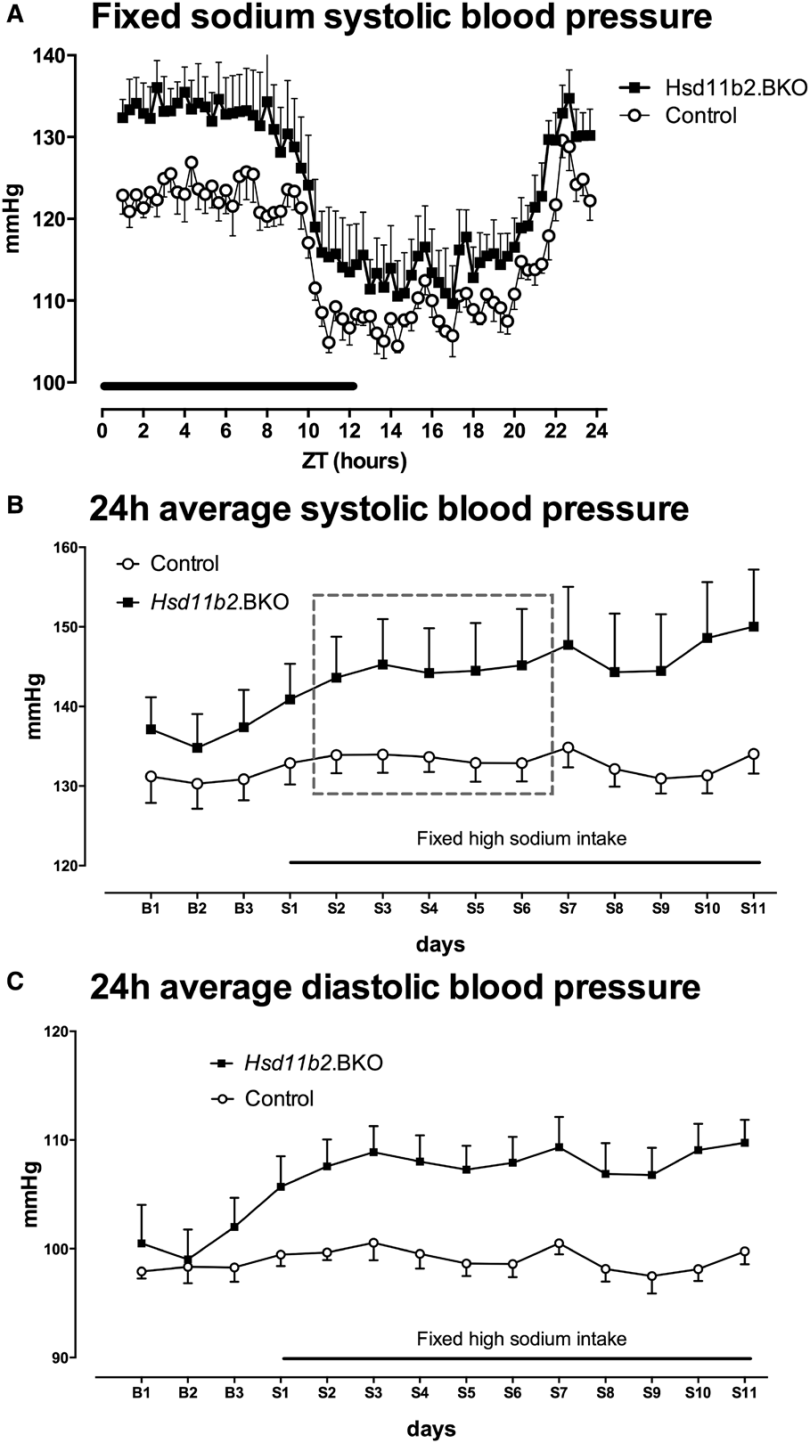
Radiotelemetry data from conscious unrestrained mice on fixed sodium intake. **A**, Systolic blood pressure measured every 20 minutes over a 24-hour period in *Hsd11b2*.BKO mice (n=6; filled squares) and controls (n=6; open circles). Data are group mean±SEM, generated by averaging each mouse over 5 consecutive days of recording. Mesor, amplitude, and acrophase were calculated by cosinor analysis (Figure I and Table II in the online-only Data Supplement) of nonaveraged data obtained over consecutive days indicated by the box. The black line indicates subjective night (7 pm to 7 am local time). Twenty-four–hour averaged systolic (**B**) and 24-hour averaged diastolic (**C**) blood pressure in *Hsd11b2*.BKO mice (filled squares) and controls (open circles) before and during a period of equivalent high-sodium feeding. Data are mean±SEM. For SBP ANOVA reported a significant effect of diet (*P*<0.0001) but not genotype (*P*=0.079); for DBP, there were significant differences for diet (*P*<0.0001), genotype (*P*=0.013), and the interaction between these main effects (*P*<0.0001). ANOVA indicates analysis of variance; DBP, diastolic blood pressure; SBP, systolic blood pressure; and SEM, standard error of the mean.

To resolve whether increased salt intake in *Hsd11b2*.BKO mice was causal or permissive for the hypertensive phenotype, the 2 groups of mice were fed an equivalent amount of sodium-rich gel-diet. The average sodium intake was 4619±121 μmol/24 h in *Hsd11b2*.BKO mice and 4790±215 μmol/24 h in controls (n=6 per group. *P*=0.452). High sodium feeding significantly increased SBP (Figure 3A and 3B) and DBP (Figure 3C) in *Hsd11b2*.BKO mice. The amplitude of the 24-hour SBP rhythm was also significantly increased (*P*=0.006; Table I in the online-only Data Supplement). Heart rate was not different between genotypes, but high salt intake reduced the amplitude of the 24-hour rhythm significantly in *Hsd11b2*.BKO mice (*P*=0.044; Table I in the online-only Data Supplement).

Blood pressure in control mice was not affected by high salt intake, indicating that the C57BL6/J background strain was not intrinsically salt sensitive. This salt resistance in the control animals means that the salt-sensitive hypertension of *Hsd11b2*.BKO mice cannot just reflect increased salt appetite. The data suggest that central homeostatic response to salt intake becomes abnormal following deletion of 11βHSD2 in the brain. This does not reflect abnormalities in systemic corticosteroid production: aldosterone and corticosterone excretion were similar in both genotypes under high-salt conditions (Table II in the online-only Data Supplement).

### Effect of Oral Dexamethasone

**Figure 4.**
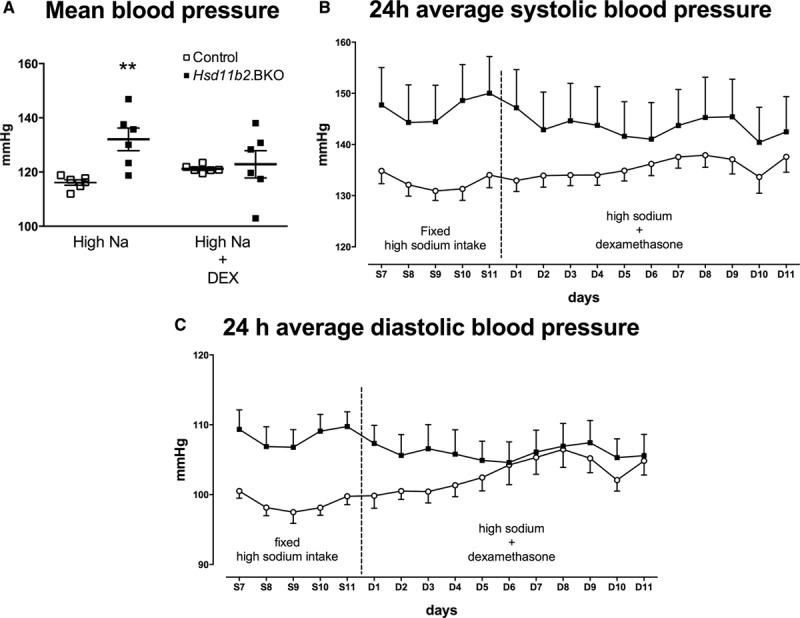
Effect of dexamethasone on blood pressure. **A**, Mean arterial blood pressure averaged over the final 5 days of the high sodium and high sodium with dexamethasone periods in *Hsd11b2*.BKO mice (n=6; filled squares) and controls (n=6; open squares). Individual mice are shown along with the group mean±SEM. Two-way ANOVA reported a significant effect of genotype (*P*=0.015) but not of treatment (*P*=0.542); the interaction between main effects was significant (*P*=0.044). Two comparisons were made, between genotypes before and after DEX treatment. **P<0.01 by Holm-Sidak post test. Twenty-four–hour average systolic (**B**) and 24-hour average diastolic (**C**) blood pressure in *Hsd11b2*.BKO mice and controls over the course of the experiment. Data are mean±SE. For both SBP and DBP, 2-way ANOVA reported a significant effect of dexamethasone (*P*<0.0001) and genotype (*P*<0.0001) and a significant interaction between the main effects (*P*<0.0001). ANOVA indicates analysis of variance; DBP, diastolic blood pressure; DEX, dexamethasone; SBP, systolic blood pressure; SE, standard error; and SEM, standard error of the mean.

Deficiency of 11βHSD2 allows MR to be activated by endogenous glucocorticoid. DEX suppression of the hypothalamo-pituitary-adrenal axis, which markedly reduces cortisol levels, can be used to treat patients with AME. DEX suppressed corticosterone (the endogenous glucocorticoid in rodents) in both *Hsd11b2.*BKO mice and controls, and, after 5 days of treatment, the genotypic difference in mean blood pressure was no longer apparent (Figure 4A). However, unequivocal interpretation of these data is challenging, because, as expected, DEX increased SBP (Figure 4B) and DBP (Figure 4C) in control mice.

### Hypertension Is Not Caused by Sodium Retention

The effect of increased salt intake on renal sodium excretion was assessed in a separate cohort of mice (n=6 for each genotype), fed first the basal salt diet (0.1% sodium) diet, followed by the high-salt (1% sodium) diet. Basal sodium intake averaged 420±15 μmol/24 h in *Hsd11b2*.BKO mice and 397±20 μmol/24 h in controls: urinary sodium excretion was not different between genotypes (Figure VIA in the online-only Data Supplement). During the high-salt phase, average sodium intake again increased 10-fold in both control (4810±177 μmol/24 h) and *Hsd11b2*.BKO (4335±240 μmol/24 h) mice and was not significantly different between the 2 groups (*P*=0.143; unpaired *t* test). Urinary sodium excretion was significantly higher in *Hsd11b2*.BKO mice than in controls during this period (Figure VIA in the online-only Data Supplement), suggesting that hypertension was not attributable to renal sodium retention.

Basal urine flow rate was slightly higher in *Hsd11b2*.BKO mice than in controls, and the diuresis prompted by high-sodium feeding was significantly greater in *Hsd11b2*.BKO mice (Figure VIB in the online-only Data Supplement). Dietary sodium feeding was not associated with marked changes in hematocrit in either genotype (Table II in the online-only Data Supplement). Overall, these data indicate that hypertension was not caused by absolute plasma volume expansion following sodium retention.

The high-sodium diet induced hypokalemia in *Hsd11b2*.BKO mice (Table II in the online-only Data Supplement). This did not reflect a change in dietary potassium intake, which was consistent throughout the study and not different between genotype. Given the exaggerated salt-induced diuresis in *Hsd11b2*.BKO mice, we anticipated that urinary potassium losses would account for potassium depletion. Although urinary potassium excretion was indeed higher in *Hsd11b2*.BKO than in controls, this difference was observed under both dietary regimens and not increased during the high-sodium feeding (Figure VIC in the online-only Data Supplement).

### Enhanced Pressor Effect of Phenylephrine and Impaired Baroreflex Gain in *Hsd11b2.BKO* Mice

**Figure 5.**
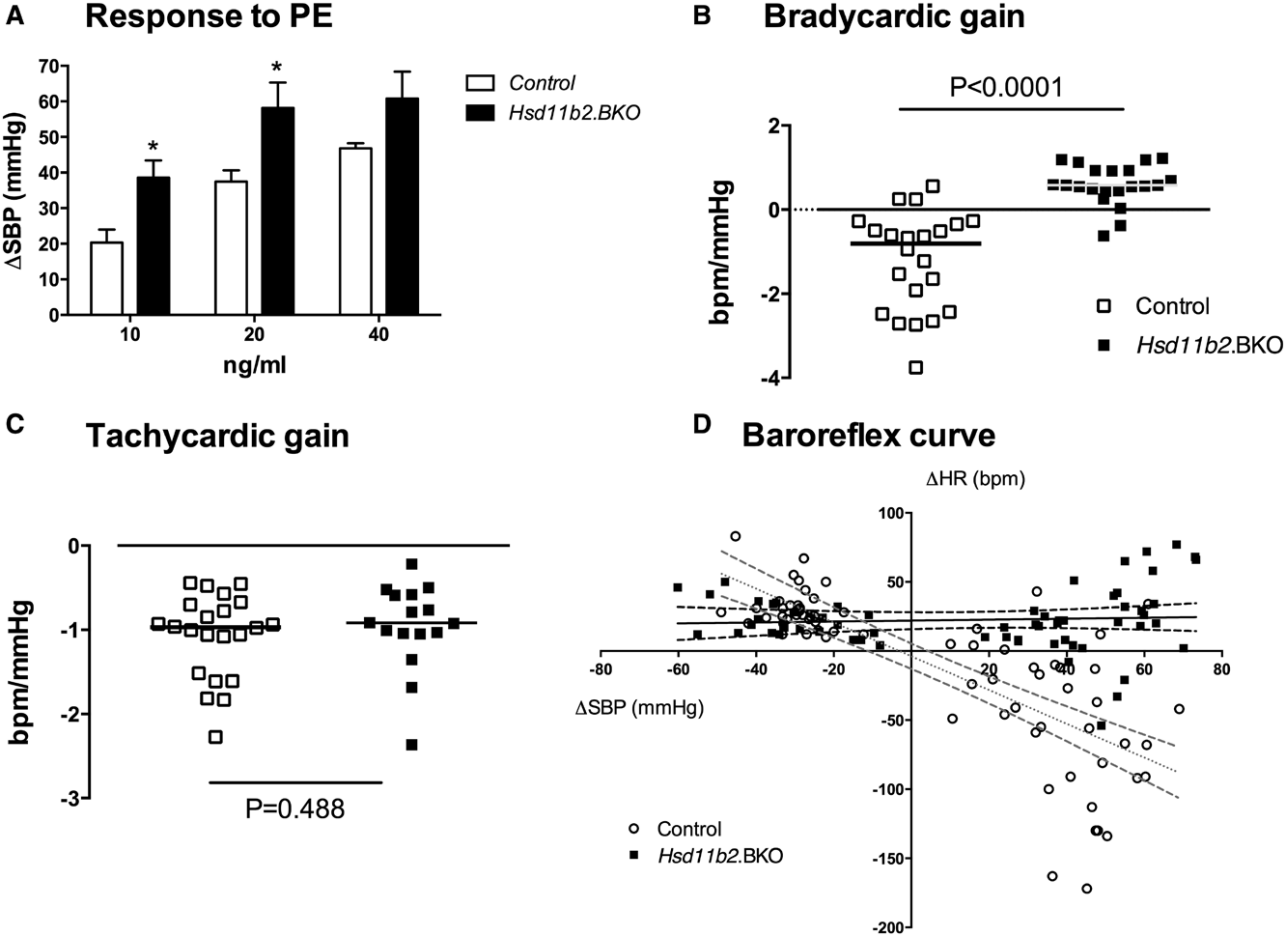
Baroreceptor reflex function. The baroreflex was measured pharmacologically in anesthetized *Hsd11b2*.BKO mice (filled squares; n=10 mice/63 responses) and controls (open circles; n=9 mice; 71 responses) mice. **A**, The mean change in systolic blood pressure (ΔSBP) in response to intravenous injection of phenylephrine. Two-way ANOVA reported a significant effect of dose (*P*<0.0001) and genotype (*P*=0.0002). Planned comparisons were made comparing each dose between genotypes. *P<0.05. The baroreflex gain during intravenous injection of phenylephrine (**B**) and during intravenous injection of sodium nitroprusside (**C**); individual data points are shown and the median compared by Mann-Whitney test, with *P* values as indicated. **D**, The baroreflex curve showing individual data points for the change in heart rate (ΔHR) in response to induced changes in systolic blood pressure (ΔSBP). There was a significant difference (*P*<0.0001) between genotypes by Linear regression analysis. ANOVA indicates analysis of variance; and PE, phenylephrine.

The salt-sensitive hypertension in *Hsd11b2*.BKO mice was not associated with a compensatory fall in heart rate, but the amplitude of the 24-hour cycle of heart rate was significantly reduced, suggesting impaired autonomic cardiac control. The NTS is an important site of baroreflex integration, and we therefore assessed directly the bradycardic response to an acutely applied pressor stimulus. In *Hsd11b2.*BKO mice maintained on a 0.1% salt diet, the pressor response to phenylephrine was significantly enhanced (Figure 5A), and the bradycardic baroreflex gain was significantly attenuated (Figure 5B). Reflex tachycardia response to sodium nitroprusside was similar in both genotypes (Figure 5C), as was the net fall in SBP. Overall, *Hsd11b2.*BKO mice displayed an asymmetrical attenuation of the baroreceptor reflex curve (Figure 5D; *P*<0.0001). Similar results were obtained in a separate cohort of *Hsd11b2*.BKO mice and controls maintained on a 1% sodium diet for 7 days (Figure VII in the online-only Data Supplement). There was no significant effect of increased dietary salt intake on baroreflex function in control mice. *Hsd11b2*.BKO mice displayed and impaired bradycardic baroreflex gain. This defect was not exaggerated by dietary salt loading.

## Discussion

Reduced 11βHSD2 activity causes a spectrum of hypertension-associated disease. Its most severe form, AME, can be rescued by renal transplantation,^8,29^ suggesting that high blood pressure follows the kidney.^9^ However, 11βHSD2 is also expressed in the brain,^17^ restricted to a subset of neurons in the NTS in the adult mouse.^20^ We used a Cre-Lox strategy to conditionally delete *Hsd11b2* in the brain, reducing expression in the NTS by >90%. We found that 11βHSD2 in the brain normally exerts significant influence over sodium homeostasis and blood pressure control, independent of renal function. We identified 3 important phenotypes in *Hsd11b2.*BKO mice: (1) an innate salt appetite, blocked by MR antagonism; (2) salt sensitivity of blood pressure, independent of salt appetite and sodium retention; and (3) an exaggerated pressor response to α-adrenoreceptor activation and an impaired reflex bradycardia.

### Central Deletion of 11βHSD2 and Salt Appetite

Negative salt balance evokes an instinctive salt-seeking behavior. The central pathways for this physiological response are not fully elucidated, but 11βHSD2-expressing neurons in the NTS are selectively activated by sodium depletion and rapidly inactivated when salt appetite is satiated.^18^ *Hsd11b2.*BKO mice had a strong salt appetite in the absence of sodium/volume depletion or systemic aldosterone excess. This underscores the concept that local corticosteroid levels in the brain influence the physiological control of sodium homeostasis. Genetic defects in central MR signaling would act synergistically with those in the distal nephron to amplify hypertension.

Systemic administration of an MR antagonist was an effective treatment but did not completely abolish salt appetite in *Hsd11b2*.BKO mice. Spironolactone is a competitive antagonist of MR and, although our method of delivery achieves high plasma concentrations of the active metabolite, canrenone,^25^ the levels reaching the NTS may be lower.^30^ Nevertheless, similar dosing regimens provide neuroprotection after cerebral ischemia in mice,^31^ and oral administration of low-dose spironolactone decreases sympathetic drive and improves baroreflex function in rats with heart failure.^32^ This suggests that central MR can be effectively blocked by systemic spironolactone, and the incomplete rescue of salt appetite in the current study may suggest that additional pathways contribute in the *Hsd11b2*.BKO mice. Central angiotensin II promotes thirst and, to a lesser extent, sodium appetite, particularly in response to sodium depletion or hypovolemia.^33^ Because water intake was not different between genotypes, we discount a major role for angiotensin II in the salt appetite of the *Hsd11b2.BKO* mice.^33^ In epithelia, MR and the glucocorticoid receptor may interact to regulate aldosterone-induced transport proteins such as ENaC.^34,35^ Indeed, we found that the salt sensitivity of the *Hsd11b2* heterozygote mouse could be blocked by glucocorticoid receptor antagonists.^13^ Whether glucocorticoid receptor contributes to salt sensitivity in *Hsd11b2*.BKO mice is not known. Although glucocorticoids are not directly natriorexigenic, they potentiate the salt appetite induced by mineralocorticoids by increasing MR expression in the brain.^36^

### Central Deletion of 11βHSD2 and Salt-Sensitive Blood Pressure

An important observation in our study was the salt-resistant blood pressure of the control mice. Thus, with the enzymatic barrier protecting MR intact, blood pressure is not affected by large (3-fold) increases in sodium intake; if the barrier is broken this same sodium load induces a rapid and sustained hypertension. The influence of 11βHSD2-positive neurons in the NTS therefore extends beyond the regulation of salt appetite by normally preventing large fluctuations in dietary salt intake from exerting corresponding changes to blood pressure.

Unlike humans,^6^ in mice^10^ or rats^37^ with global 11βHSD2 deficiency, deletion in the brain alone is not sufficient to change basal blood pressure, and the additional insult of a sustained high sodium intake is required for hypertension. The nature of this interaction is not yet defined. High salt intake was necessary but not sufficient for the hypertensive response, a situation analogous to the pressor effect of intracerebrovascular aldosterone infusion, which is sensitized by, but not exclusively dependent on, sodium intake.^38^

What activates MR to induce salt sensitivity? Aldosterone synthase is expressed in rat brain,^39^ and aldosterone is synthesized centrally.^40^ However, this is not the case in mouse and human brains,^41,42^ and salt sensitivity in *Hsd11b2*.BKO mice is unlikely to reflect central aldosterone excess. Corticosterone and the neurosteroid precursor deoxycorticosterone are plausible alternatives. Indeed, oral DEX attenuated the blood pressure differential between genotypes. However, it is difficult to interpret these data because DEX did not actually reduce blood pressure in *Hsd11b2*.BKO mice. Instead, DEX increased blood pressure in control animals but not in *Hsd11b2*.BKO mice. It is likely that the peripheral pressor effects of excess DEX offset the reversal of central salt sensitivity, making the overall benefit for blood pressure in *Hsd11b2.*BKO mice modest.

### Central Deletion of 11βHSD2 and Peripheral Blood Pressure Control

Salt sensitivity was not associated with sodium retention; urinary sodium excretion was higher in *Hsd11b2*.BKO mice than in controls during the dietary salt challenge. The regulation of blood pressure by aldosterone-target neurons in the NTS appears independent of kidney function, suggesting that MR-dependent hypertension may have a substantial neurogenic component.^22,43^ In other salt-sensitive models, increased central sympathetic drive and increased peripheral resistance sustain hypertension.^44–46^ The salt-induced increase in DBP and heightened pressor responsiveness to α-adrenoreceptor agonism in *Hsd11b2*.BKO mice are consistent with this hypothesis. Similarly, salt-induced hypokalemia in the absence of potassium wasting may suggest redistribution of potassium into the intracellular compartment following sympathetic activation.^47^ An impaired baroreflex would release tonic inhibition of sympathetic nerve activity, increasing sympathetic drive to the peripheral vasculature. In *Hsd11b2.BKO* mice, the impairment was asymmetrical, and the ability to buffer a pressor response was compromised. Similar observations are found in healthy humans following systemic aldosterone infusion^48^ and in patients with mild congestive heart failure,^49^ and contribute to increased cardiovascular risk in these patients.^50^

### Summary and Perspectives

Our study demonstrates a unifying link between activation of MR in the NTS, salt appetite, and blood pressure control. In the absence of a physiological stimulus to consume salt, this arc is maladaptive and causes salt-sensitive hypertension. These same molecular pathways regulate renal salt reabsorption. Thus, global mutations in key genes will give a double hit for hypertension by increasing the behavioral drive to consume sodium and impairing the ability of the kidney to excrete this salt. Given that global sodium intake is habitually high, this integrated framework of sodium homeostasis is highly relevant and suggests that MR antagonists could be used to improve compliance to dietary sodium restriction in the treatment of cardiovascular disease.

## Sources of Funding

Drs Evans and Ivy were funded by British Heart Foundation 4-year PhD studentships (FS/07/063/24075; FS/11/78/29328). Dr Christensen was funded by the Lundbeck Foundation (R152-2013–14574). Dr McNairn was funded by the Medical Research Council Doctoral Training Award to The University. The authors acknowledge support from The British Heart Foundation Center of Research Excellence Award (RE/08/001), Kidney Research UK (IN11/2011), and The Wellcome Trust (WT079009).

## Disclosures

None.

## Supplementary Material

## CLINICAL PERSPECTIVE

For the majority of people in industrialized societies, dietary salt intake habitually exceeds the recommended upper tolerable limit. This sustained high salt intake is associated with hypertension and with increased risk of cardiovascular disease. Reducing sodium intake may be beneficial for a large number of people, particularly those with hypertension or heart failure. However, compliance to restricted salt intake is poor, which may in part reflect enhanced salt appetite. The central pathways controlling salt intake are incompletely defined, but it is known that certain neurons in the brain stem are activated by salt depletion. We genetically modified mice, removing a gene in the brain stem to amplify local aldosterone signaling. Basal blood pressure and systemic electrolyte and hormonal status were not affected by this genetic modification. However, ad libitum salt intake increased 3-fold and this caused hypertension. We were able to partially block salt appetite with the mineralocorticoid antagonist spironolactone. This study demonstrates an important role for brain stem pathways in the control of sodium homeostasis and blood pressure. Mineralocorticoid antagonists could help improve compliance to restricted salt regimens during the management of cardiovascular disease.
