# Supplementary material for: Conditional Deletion of Hsd11b2 in the Brain Causes Salt Appetite and Hypertension
Source: Circulation. 2016 Apr 4;133(14):1360–70. doi: 10.1161/CIRCULATIONAHA.115.019341 (PMC4819772; doi:10.1161/CIRCULATIONAHA.115.019341)

SUPPLEMENTAL MATERIAL

Supplemental Table 1. Cosinor analysis of A) systolic blood pressure and B) heart rate in control and *Hsd11b2*.BKO mice made from recordings over 5 consecutive days in each of the baseline, *ad libitum* and fixed salt phases. Mesor (mmHg); amplitude (mmHg) and acrophase (degrees) are shown as group arithmetic mean±SE;. The P values are 2-tailed and comparisons were made using unpaired t-tests.

A) Systolic Blood Pressure

	**Control**	**P**	***Hsd11b2*.BKO**
Baseline			
Mesor	129.8±1.9	0.748	131.6±5.33
Amplitude	12.91±0.90	0.345	11.24±1.40
Acrophase	-1.25±0.05	**0.073**	-1.40±0.06
Ad lib. salt intake			
Mesor	130.6±2.0	0.096	140.1±4.5
Amplitude	10.57±0.84	**0.035**	13.60±0.83
Acrophase	-1.03±0.06	0.143	-1.22±0.09
Fixed salt intake			
Mesor	134.7±2.3	0.252	144.7±7.6
Amplitude	10.70±0.78	**0.006**	15.2±1.02
Acrophase	-0.97±0.01	0.342	-1.01±0.11

B) Heart rate

	**Control**	**P**	***Hsd11b2*.BKO**
Baseline			
Mesor	571±7	0.937	570±16
Amplitude	70±5	0.443	62±8
Acrophase	-1.21±0.07	0.222	-1.03±0.12
Ad lib. salt intake			
Mesor	472±83	0.368	555±17
Amplitude	52±3	0.814	54±9
Acrophase	-0.64±0.08	0.963	-0.64±0.06
Fixed salt intake			
Mesor	542±8	0.677	535±15
Amplitude	56±6	**0.044**	37±5
Acrophase	-0.45±0.06	0.089	-3.19±0.08

Supplemental Table 2. Plasma sodium and potassium concentration, haematocrit and urinary excretion of aldosterone and corticosterone for control and *Hsd11b2*.BKO mice maintained on either a 0.1% or 1% sodium diet. Data are mean±SEM and comparisons were made by 1-way ANOVA with Holm-Sidak *post-hoc* test used to test 3 planned comparisons with a family alpha of 0.05. *P<0.05; **P<0.01 within genotype; ††P<0.01 between genotype.

	**Control**<br><br>**n=6**	**Control**<br><br>**n=6**	***Hsd11b2*.BKO**<br><br>**n=6**	***Hsd11b2*.BKO**<br><br>**n=6**	***ANOVA P***
Diet	0.1% Na	1% Na	0.1% Na	1% Na	
P_{Na} (mmol/l)	147.0±0.7	151.0±1.2	154.3±2.0††	153.0±1.2	0.008
P_{K} (mmol/l)	4.84±0.25	4.26±1.21	4.79±0.18	3.09±0.27**	<0.0001
Hct (%)	44±2	42±1	41±2	44±1	0.293
U_{Aldo} (pmol/24h)	2.15±0.45	3.58±0.78	1.83±0.24	3.35±0.41	0.197
U_{Cort} (pmol/24h)	209±13	329±29*	196±25	294±50	0.047

Supplemental Figure 1. *Hsd11b2* mRNA abundance in the Nucleus of the Solitary Tract (NTS) micro-dissected from adult male control (n=13; open circles) and *Hsd11b2*.BKO mice (n=11; black squares). The brain was removed after cervical dislocation and the hind-brain was cut away from the forebrain and the cerebellum removed. The top half of the medial section of the hind-brain, containing the NTS was collected for extraction of total RNA. A) *Hsd11b2* mRNA abundance expressed in arbitrary units (AU) was normalised to that of hypoxanthine guanine phosphoribosyl transferase (*hprt*) in the same sample. B) The percentage reduction in *Hsd11b2* expression in *Hsd11b2*.BKO mice was calculated by normalizing to the mean expression of the control group. Individual points are shown with the group median and interquartile range. The comparisons were made using the Mann-Whitney test.

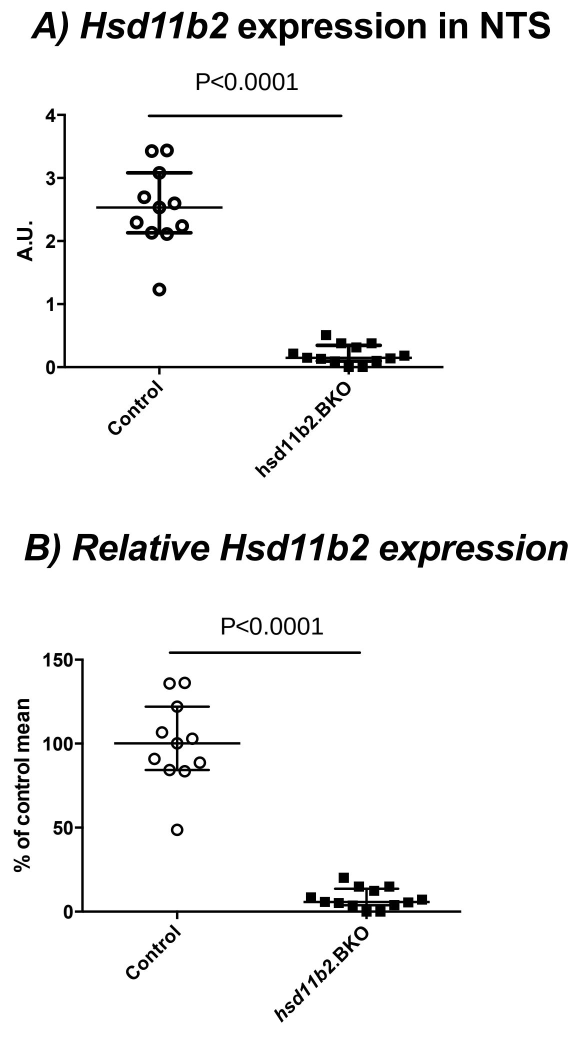

.

Supplemental Figure 2. Expression of 11βHSD2 imunoreactivity in fixed sections from A) control and B) *Hsd11b2*.BKO mouse kidney. 11βHSD2 expression was restricted to the collecting duct segments. Images (x200 magnification) of cortical collecting ducts are shown and in both genotypes, 11βHSD2 was expressed in principal cells, but not in intercalated cells. C) *Hsd11b2* mRNA abundance and D) 11βHSD2 enzyme activity in whole kidney homogenates. There were no differences between genotype analysed by Mann-Whitney test and unpaired t-test, respectively.

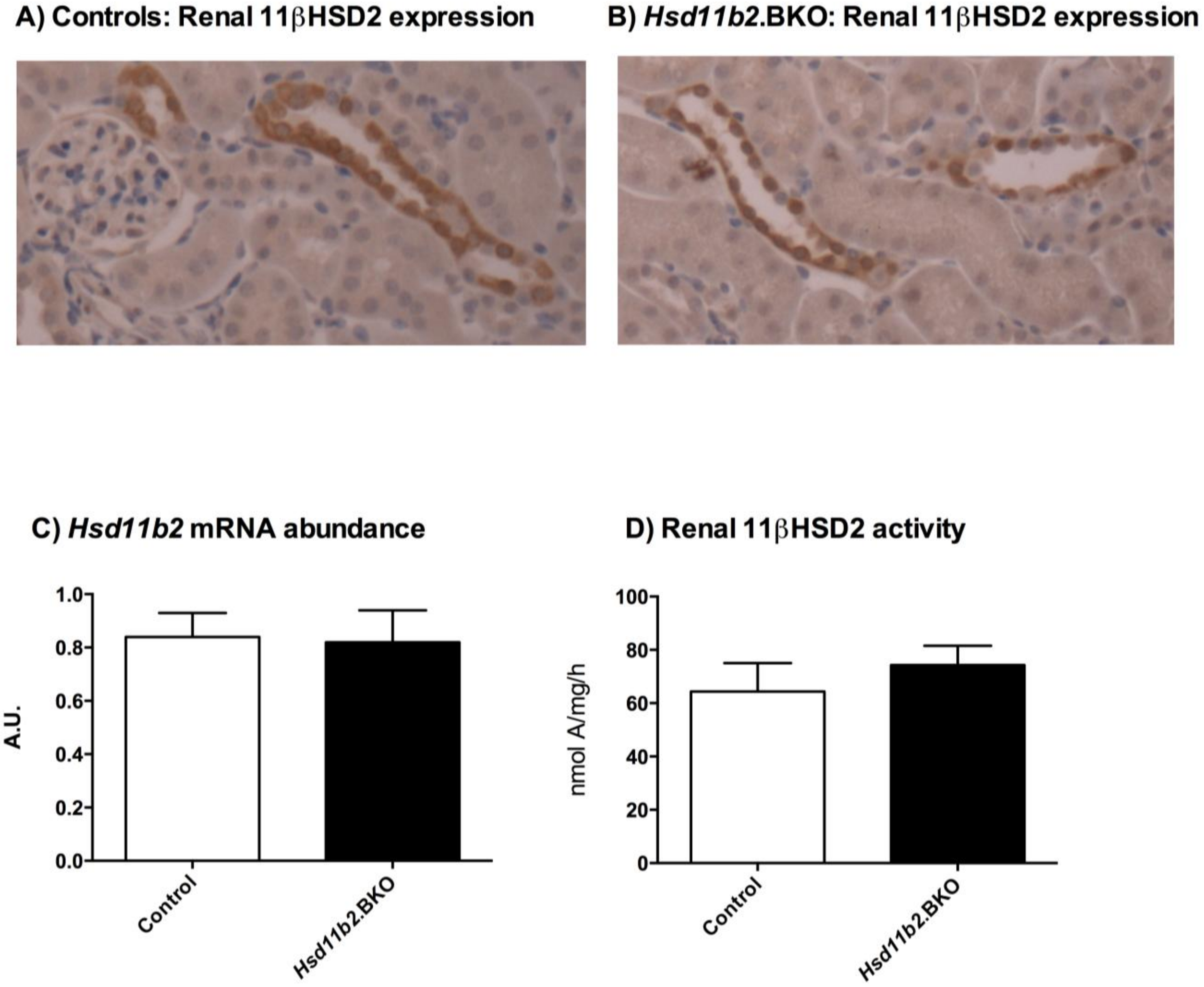

Supplemental Figure 3. A) Systolic blood pressure; B) diastolic blood pressure; and C) heart rate in control (open symbol; n=6) and *Hsd11b2*.BKO (black symbol; n=6) mice. Recordings were made by radiotelemetry over 7 consecutive days during which all mice had *ad libitum* access to standard rodent diet and d$H_2$0. The diurnal variability was assessed in each mouse over the final 4 days of recording and data combined to give a group average. Bar indicates subjective night. Data are shown as mean ± SEM.

A) Systolic blood pressure

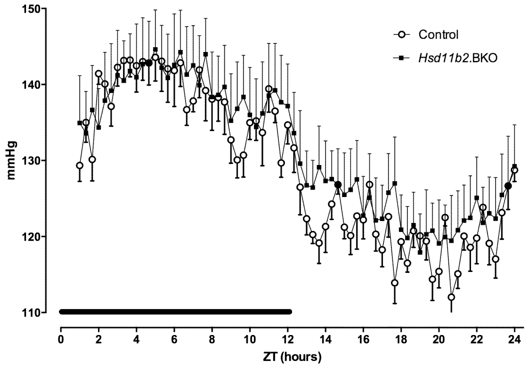

B) Diastolic blood pressure

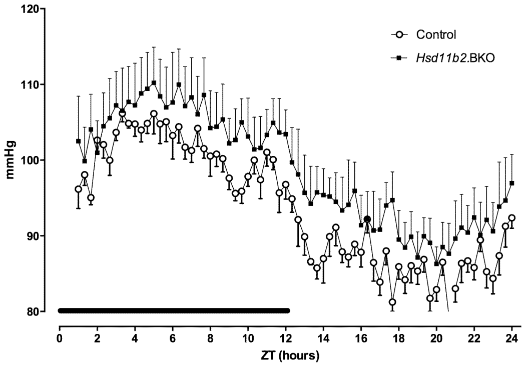

C) Heart Rate

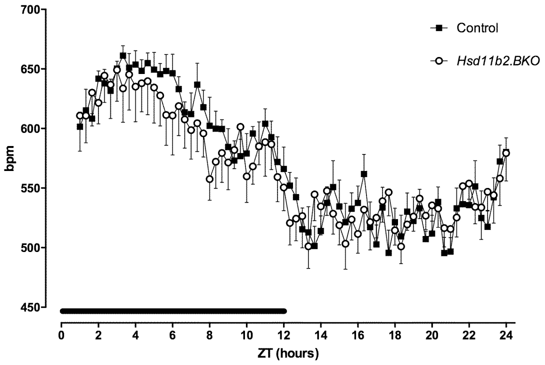

Supplemental Figure 4. The effect of salt on mesor and amplitude SBP in control (open circles; n=6) and *Hsd11b2*.BKO (black squares; n=6) mice. Group mean and SEM are shown in Supplemental Table 2. In this figure, data points (with group means ± SEM) from individual mice are shown for A) mesor and B) amplitude of SBP. Two-way ANOVA with repeated measures was used to assess the main effects of salt diet and genotype and the interaction between these two. For mesor, there was a significant effect of diet (P=0.016) and genotype (P=0.008), but not of interaction (P=0.161). For amplitude there was no significant effect of salt diet (P=0.623), but the effect of genotype was different (P=0.039) as was the interaction (P=0.015). Planned comparisons were made within genotype, as indicated.

A) Mesor time-series

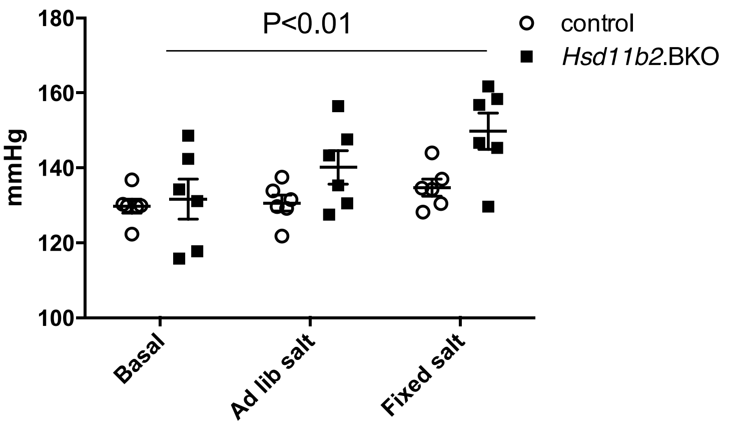

B) Amplitude time-series

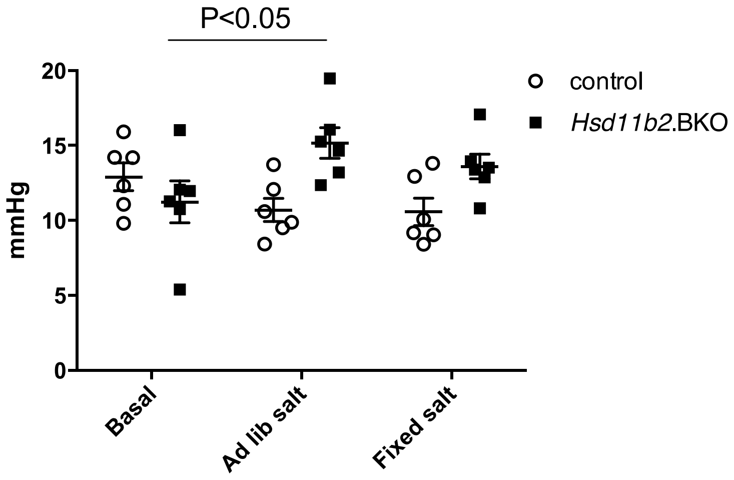

Supplemental Figure 5. Heart rate in control (open circle; n=6) and *hsd11b2*.BKO (black square; n=6) mice. Recordings were made by radiotelemetry and mice had *ad libitum* access to standard rodent diet and two drinking bottles containing dH_20 and 1.5% NaCl, respectively. Bottles were rotated every 24 hours. The diurnal variability was assessed in each mouse over the final 4 days of recording and data combined to give a group mean ± SEM. The bar indicates subjective night

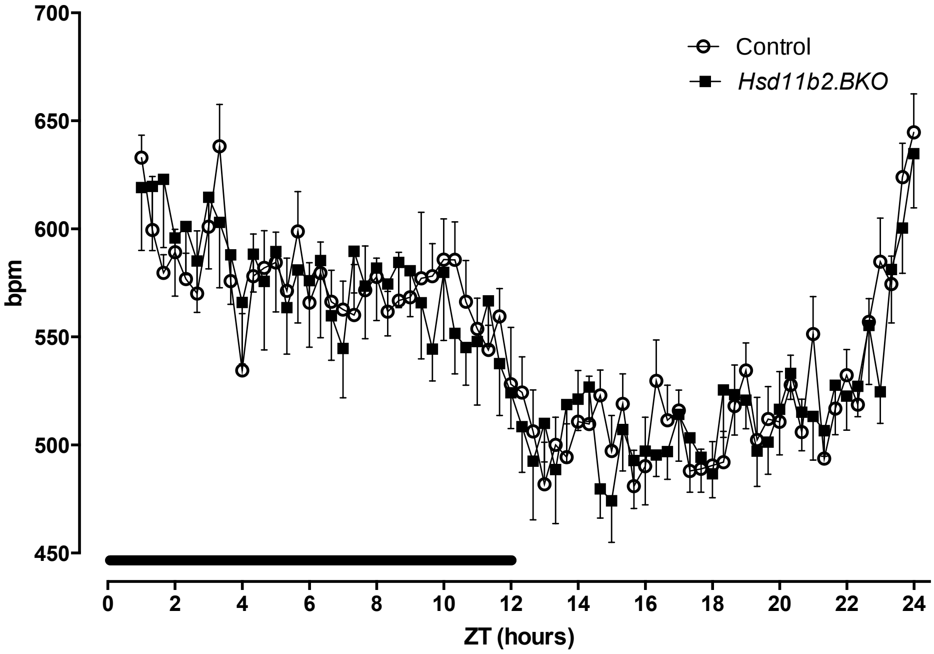

Supplemental Figure 6. A) sodium excretion, B) urine flow rate and C) potassium excretion in control mice (n=6; open circles) and *Hsd11b2*.BKO mice (n=6, black squares). Mice were fed a gel diet delivering a fixed sodium intake per day. For the first 5 days, mice received 0.1% Na diet before being fed 1% Na diet for the next 8 days. Data, normalized to body weight, are mean ± SEM. Two-way ANOVA with repeated measures was used to assess the main effects of salt diet and genotype and the interaction between these two. For sodium excretion, the effect of diet was significant (P<0.0001), the effect of genotype not significant (P=0.063) and the interaction significant (P=0.048). For urine flow rate the effect of diet (P<0.0001), genotype (P=0.004) and the interaction (P<0.0001) was significant. For potassium excretion the effect of diet (P<0.0001 and genotype (P=0.035) was significant; the interaction was not significant (P=0.837). No planned or post-hoc comparisons were made.

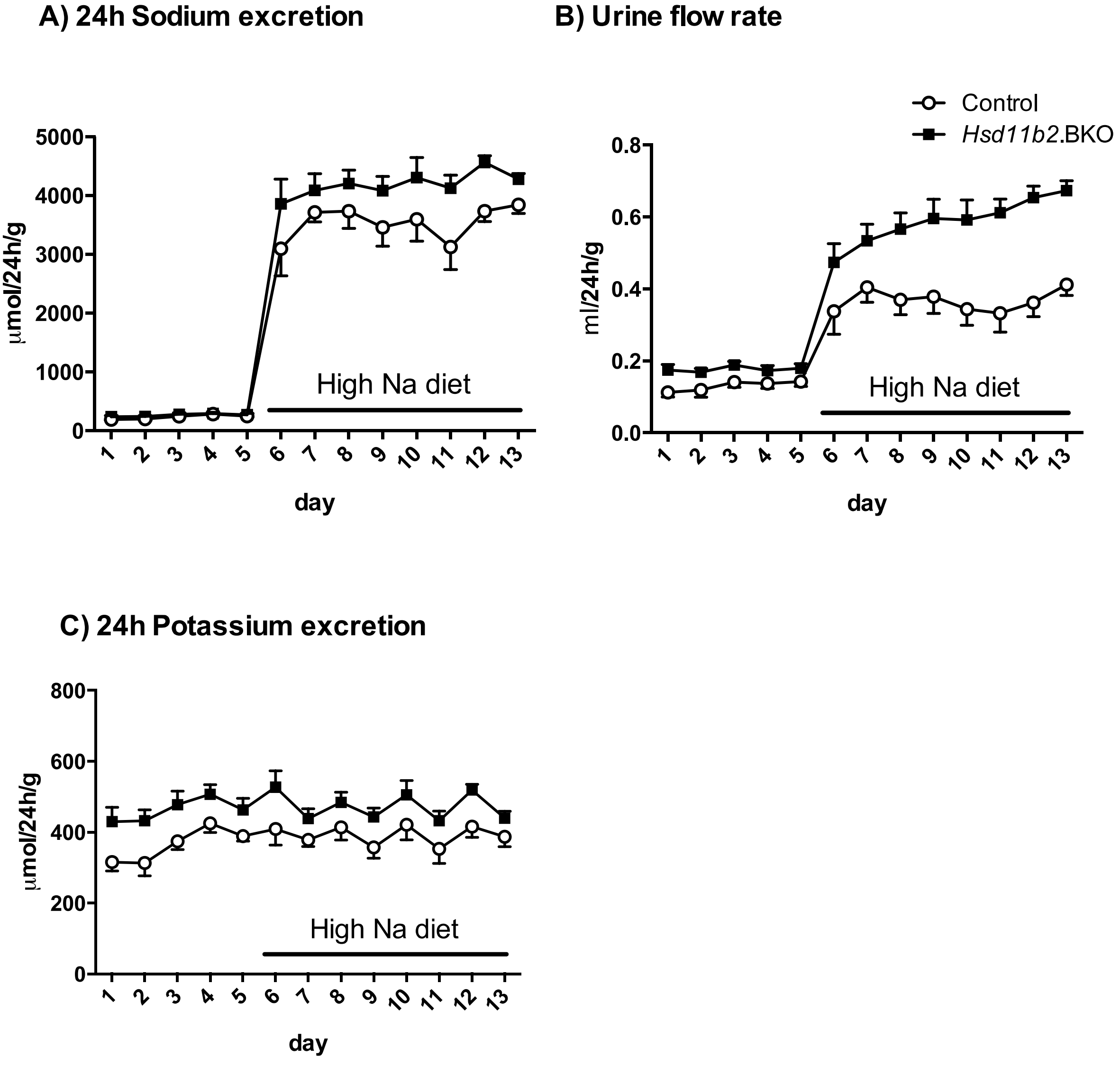

Supplemental Figure 7. Baroreceptor reflex function under high salt conditions. The baroreflex was measured pharmacologically in anaesthetized *Hsd11b2*.BKO mice (black squares; n=5 mice/59 responses) and controls (white circles; n=6 mice; 71 responses) mice after 7 days of *ad libitum* access to 2.5% salt diet. A) the baroreflex curve showing individual data points for the change in heart rate (ΔHR) in response to induced changes in systolic blood pressure (ΔSBP). There was a significant difference (P<0.0001) between genotypes bylLinear regression analysis. B) the baroreflex gain during intravenous injection of sodium nitroprusside (tachycardic gain) and during C) intravenous injection of phenylephrine (bradycardic gain); individual data points are shown, with the median and IQR. Comparisons were by Mann-Whitney test, with P values as indicated.

A) Baroreflex curve on high salt diet

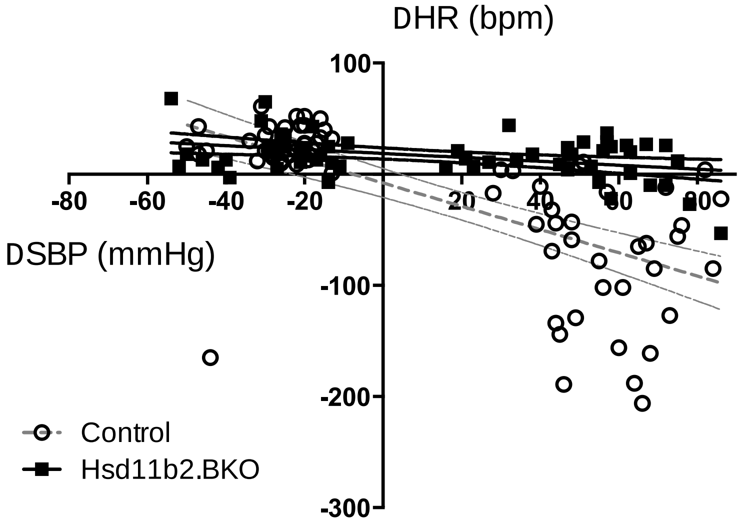

B) Tachycardic gain

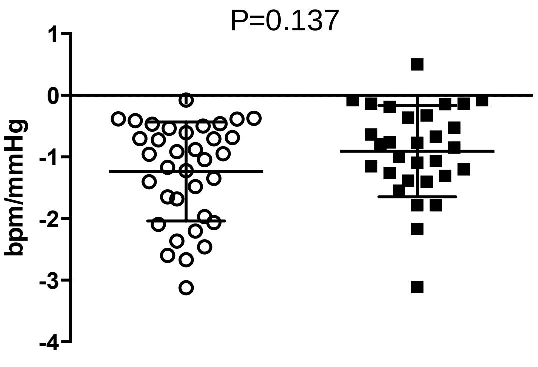

C) Bradycardic gain

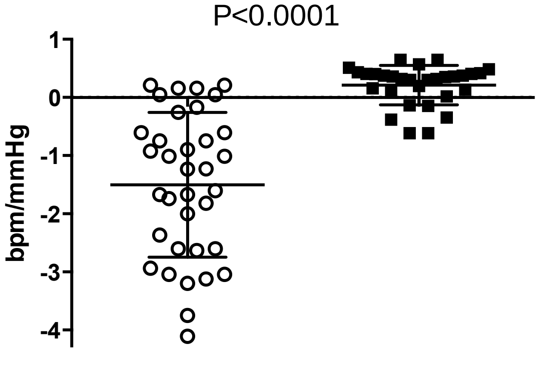

Supplement: Supplementary file 1 [file cir-133-1360-s001.pdf]
